# Functional disruption of cortical cingulate activity attenuates visceral hypersensitivity and anxiety induced by acute experimental colitis

**DOI:** 10.1038/s41598-021-81256-x

**Published:** 2021-01-22

**Authors:** Lukas Brenner, Leah Zerlin, Linette Liqi Tan

**Affiliations:** Institute of Pharmacology, Heidelberg University, Im Neuenheimer Feld 366, 69120 Heidelberg, Germany

**Keywords:** Inflammatory bowel disease, Neural circuits, Sensory processing

## Abstract

Visceral pain is a highly complex experience and is the most common pathological feature in patients suffering from inflammatory gastrointestinal disorders. Whilst it is increasingly recognized that aberrant neural processing within the gut-brain axis plays a key role in development of neurological symptoms, the underlying mechanisms remain largely unknown. Here, we investigated the cortical activation patterns and effects of non-invasive chemogenetic suppression of cortical activity on visceral hypersensitivity and anxiety-related phenotypes in a well-characterized mouse model of acute colitis induced by dextran sulfate sodium (DSS). We found that within the widespread cortical network, the mid-cingulate cortex (MCC) was consistently highly activated in response to innocuous and noxious mechanical stimulation of the colon. Furthermore, during acute experimental colitis, impairing the activity of the MCC successfully alleviated visceral hypersensitivity, anxiety-like behaviors and visceromotor responses to colorectal distensions (CRDs) via downregulating the excitability of the posterior insula (PI), somatosensory and the rostral anterior cingulate cortices (rACC), but not the prefrontal or anterior insula cortices. These results provide a mechanistic insight into the central cortical circuits underlying painful visceral manifestations and implicate MCC plasticity as a putative target in cingulate-mediated therapies for bowel disorders.

## Introduction

Inflammatory bowel diseases (IBD), including ulcerative colitis and Crohn’s disease, are common incurable lifelong conditions characterized by unpredictable recurring episodes of gastrointestinal tract inflammation, severe abdominal pain, rectal bleeding and diarrhea. Clinical data suggests that IBD occurrence is rising worldwide, with currently an estimated 2.5–3 million affected people in Europe alone^1^, thereby accounting for a substantial health care and socioeconomic burden.

In addition to the pathophysiological manifestations of the disease, IBD patients also frequently suffer from psychological and social burden, leading to a poorer quality of life^2^. Indeed, persistent anxiety- and depression-related symptoms are highly prevalent amongst IBD sufferers and such psychological stress, in turn, is likely associated with an exacerbation of IBD symptoms^3,4^. Similar behavioral disturbances and cognitive impairments have also been observed in animal models of IBD^5,6^, making them a useful tool in understanding the pathophysiological changes that arise in the brain in response to experimental intestinal inflammation.

Recently, increasing evidence demonstrates a link between intestinal inflammation and altered brain function^7–9^ in particular concerning neurological and behavioral changes, leading to the well-accepted notion of the importance of the gut-brain axis in health and disease. For instance, bowel inflammation has been shown to lead to cytokine-induced disruption of adult neurogenesis in the hippocampus^10^, changes in inflammatory-related marker expression and microglia activation levels in various cortical and subcortical regions^11–13^ as well as dysregulation of the hypothalamic–pituitary–adrenal axis^14^, all which can lead to modified brain excitability.

Despite severe pain being the chief hallmark of IBDs^15^, most attention has been focused on the peripheral and immunological features of IBD pathology whilst studies of supraspinal top-down modulation on the development and maintenance of visceral pain in IBDs has lagged behind. Clinical imaging and neuroanatomical analyses have reported abnormalities in evoked brain responses, resting-state activity and connectivity, as well as gray and white matter properties of IBD patients^16^. Interestingly, differences in structural properties of the cingulate and insula cortices were reported between painful and painless IBD^17^, suggesting that abnormal sensory processing in these cortical regions is highly correlated with the severity of pain symptoms in IBD patients. In experimental models, the impact of colitis on central excitability, pain and comorbidities has only recently begun to be studied^5^ and further investigations are urgently warranted especially since current pharmacological therapies of painful IBDs frequently remain ineffective or have adverse side effects.

Amongst the multiple cingulate parcellations, the mid-cingulate cortex (MCC) subregion partakes in numerous brain functions such as sensory processing, attention, cognition and has more recently been shown to participate in acute and chronic pain processing^18–21^. However, the functional link between MCC and its associated circuits in the modulation of visceral pain and comorbid anxiety particularly during gut inflammation, has not been previously explored.

In the current study, we investigated the effects of cortical neural activity modulation on the processing of noxious visceral stimulations in experimental colitis. We used the well-characterized mouse model of acute IBD induced by oral administration of dextran sulphate sodium (DSS) in drinking water, which results in pathophysiological features such as disrupted intestinal epithelial barrier, increased intestinal permeability and macrophage activation often reported in IBD patients^22,23^. Further, using a combination of chemogenetic approach (with designer receptor exclusively activated by designer drugs; DREADD), behavioral paradigms and molecular mapping analyses using c-Fos (marker for neuronal activation), we show that activity in the mid-cingulate cortex (MCC) is highly recruited during visceral nociceptive processing under both normal and inflamed gut conditions and that functional suppression of MCC excitation successfully attenuates colitis-induced visceral mechanical hypersensitivity and an anxiety-like phenotype via dampening excitability in specific cortical networks.

## Methods

### Animals

Adult male C57Bl6 mice (25–30 g, 12–16 weeks old) were used in this study. Animals were housed in groups and provided with food and water ad libitum on a 12 h light/dark cycle. The experimental procedures were approved by and performed according to the ethical guidelines and welfare regulations set by the local governing body (Governmental Council in Karlsruhe, Germany; approval numbers 35-9185.81/G206/18) and are in compliance with the ARRIVE guidelines.

### Surgical procedures

#### Stereotactic injections

The animals were deeply anaesthetized by an intraperitoneal injection (*i.p.*) of a mixture of fentanyl (0.05 mg/ml), medetomidine hydrochloride (1 mg/ml) and midazolam (5 mg/ml) (ratio of 4:6:16 respectively, 0.7 µl per gram of body weight). The head fur was shaved off and lidocaine (10%) was applied to the skin surface. A medial skin incision was made to expose the skull and holes (200 µm in diameter) were drilled above the regions of interests. Stereotactic injections of recombinant adeno-associated virus (rAAVs) were performed along the following coordinates to infect the left and right MCC (relative to Bregma): 0.02 mm anterior, 0.2 mm lateral, 1 mm depth; 0.22 mm posterior, 0.2 mm lateral, 1 mm depth; 0.46 mm posterior, 0.25 mm lateral, 1 mm depth and; 0.7 mm posterior, 0.25 mm lateral, 1 and 0.6 mm depths, according to the mouse brain atlas^24^. The mice were randomly allocated to receive either rAAV5-synapsin-hM4Di-mCherry or rAAV5-synapsin-mCherry (identifier v84, v116; purchased from University of Zurich viral vector facility, Switzerland; 0.05 µL over 5–10 min at each site). Sequence information for both plasmids is available at https://www.addgene.org. Lidocaine (10%) was applied to the sutured skin surface and post-surgical analgesia was given. Animals were left to recover in heated cages and kept for 3 weeks to achieve optimal viral expression. Post-hoc analysis of the expression of the rAAVs within the MCC were undertaken for each animal at the end of the experiment, animals exhibiting viral expression that spread out of the MCC boundaries were not included in this study.

#### Implantation of telemetry sensors

Animals were deeply anaesthetized in the same manner as described above. The lower abdominal fur was shaved and a midline skin incision (3–4 cm) was made. A small pocket between the skin and abdominal muscles was made in the right flank with blunt forceps and a sterile miniature telemetry implant (ETA-F10 XSmall Implant, ADInstruments, United Kingdom) was secured in the pocket. The ends of the lead wires were sutured to the superficial layers of the lower abdominal muscles 1 cm left of the midline. The skin was then sutured and animals were given post-surgical analgesia and left to recover for 10 days before behavioral experiments.

### Induction of colitis

To induce colitis, the experimental animals were given ad libitum access to dextran sulfate sodium (DSS, 40 kDa, 1.5–2%, catalog 42867, Merck KGaA, Germany) in tap water for 5 days (Day 1 to 5), followed by normal tap water for an additional 4 days (Day 6–10). Control animals received normal tap water. Throughout the 10 days, animals were monitored daily for weight changes, disease activity and symptom severity indices. The disease activity index was calculated as a total score from three measures: weight loss, stool consistency and visible fecal blood (0 = no changes; 1 =  < 5% weight loss; 2 = 6–10% weight loss or soft stools or detected fecal blood; 3 = 11–20% weight loss or loose stool or detected fecal blood; 4 =  > 20% weight loss or watery stool or gross fecal bleeding. The presence of fecal blood was tested with hemoccult testing kits (Beckman Coulter Inc, United States). A symptom severity index was additionally scored (0 = no symptoms, 1 = mild, 3 = severe) based on inactivity, posture, fur condition, breathing rate, crusty eyes, shivering and alertness. Animals were euthanized immediately if a total score of 4 was reached. Behavioral experiments were carried out between Day 6–10 when the colitis was well developed.

### Behavioral tests

All behavioral measurements were carried out during the light cycle of the animals.

#### von Frey test

Fur from the abdominal region of the animals was removed 2 days prior to measurements. Animals were acclimatized to the behavioral setup with several daily 60 min sessions. Testing of the mechanical sensitivity of the lower abdominal region was subsequently carried out in acclimatized animals via applications of von Frey filaments with increasing forces (0.008 to 1 g) and abdominal withdrawal frequencies were recorded (5 applications per filament applied at 3 min intervals). Measurements were made prior to an *i.p.* injection of clozapine-N-oxide (CNO, 2 mg/kg body weight, LFT-C4759, Biomol GmbH, Germany) and 60 min post-CNO injection. The 40% mechanical threshold corresponds to the force at which the animal withdrew at least 40% (i.e. at least 2 out of 5 applications). The experimenter was blinded to the identity of the animals.

#### Open field exploratory test

The open field test was performed with a 44 × 44 cm box under dim light conditions (40–60 lx). As this is a novel exploratory test, we performed the test in 4 cohorts of animals. Two groups of animals (mCherry- and hM4Di-expressing) were assessed in the open field arena under normal non-inflamed conditions. To evaluate the impact of MCC inhibition on colitis-induced anxiety-related behaviors, another two groups of animals (mCherry- and hM4Di-expressing) received an *i.p.* injection of CNO (2 mg/kg) on DSS Day 10 and were then assessed in the open field arena 60 min after the injection. Each mouse was placed in the center of the box and its exploration activity was recorded for 10 min via the ANY-maze tracking software (version 6.1, Stoelting Co., Ireland). A middle square comprising of 20 × 20 cm was designated as the center zone. Parameters such as total distance ran, mean speed and time spent in the center zone were analyzed.

#### Electromyogram (EMG) recordings and colorectal distensions

Abdominal muscle EMG readings were acquired wirelessly in freely moving animals via the LabChart software (version 8) using the biopotential telemetry foundation system (ADInstruments, United Kingdom). The EMG readings were used as a measure of abdominal contractions that represented the extent of visceral pain to colorectal distension (CRD) in vivo. Under a brief anasesthesia (< 30 s, 2% isoflurane), a lubricated balloon (2 mm width when deflated) was gently inserted through the anal opening into the colon (3 cm depth) of telemetry sensor-implanted animals and secured to the base of the tail with tape. The balloon was made of thin polyethylene and was attached to a soft plastic tubing (1 mm in diameter) that was connected to a digital pressure meter (Greisinger GmbH, Germany) to monitor the pressure in the balloon. Pressure readings (mmHg) were recorded electronically and time-logged to the EMG recordings. After the balloon insertion, the animals were placed on the telemetry receiver plates to recover from the brief anesthesia for at least 15 min before the start of the recordings. Basal activity (no distension) was recorded for 15 min, subsequently, air supplied from a syringe was introduced into the balloon at 20, 40, 60 and 80 mmHg. Three trials of each pressure were applied, each trial consisted of a 20 s distension application followed immediately by a 5 min resting intervals (with no distension). Animals were acclimatized to the balloon and recording setup for two 30 min sessions prior to the recording. Measurements recorded pre- versus post-CNO injections were carried out separately over consecutive days to minimize stress to the animals.

The abdominal contractions resulting from the balloon distensions can be visualized through bursts of EMG activity that were continuously collected at a frequency of 2000 Hz. For the EMG signal quantification, a mV threshold (8 × the standard error of the average mW) was first determined from a 10 s period of basal activity immediately prior to each distension. The threshold was then applied to the EMG signal during the distension trial and the number of EMG spikes that occurred above this threshold were analyzed. EMG activity was assessed during the CRD (20 s) and during the inter-stimulus interval (300 s) between each CRD application. Body activity counts per second were also recorded via the telemetry sensor and represented as an integral of the total activity recorded during the CRD applications.

### Histology

Animals were transcardially-perfused with phosphate buffered saline (PBS) (0.1 M, pH 7.4) followed by 4% paraformaldehyde (PFA). The entire colon was subsequently removed and post-fixed overnight at 4 °C in 4% PFA before being transferred to 0.5% PFA. Prior to cutting with the cryostat, the colon was flushed gently with PBS and 0.5–1 cm of the distal end of the colon tissue was cut and placed in a solution of 30% sucrose in PBS for 24 h. The tissue was embedded in tissue freezing medium, cut at 20 µm thick sections at − 25 °C and kept on slides at − 20 °C until use. The slides were defrosted, then dipped in cold acetone and left to dry for 15 min prior to staining with hematoxylin and eosin for histological evaluation.

#### Histology scoring

The colon tissues were scored in a blinded manner according to the previously published scheme for histological damage^25^ with minor modifications. The following parameters were analyzed and scored according to the extent of damage: crypt architecture (normal: 0—loss of entire crypt architecture: 5), inflammatory cell infiltrates (normal: 0—strong infiltration in large areas: 5), goblet cell density (normal: 0—goblet cell depletion: 5), local edema (normal: 0—severe edema in mucosal and submucosal regions and/or muscle thickening: 5).

### Fos immunofluorescence

Animals were given CRDs three times (80 mmHg, 20 s each) at 5 min intervals in the same manner as described above and thereafter transcardially-perfused with PBS followed by 4% PFA 60 min post-stimulation. The brain was removed after perfusion and further post-fixed overnight in 4% PFA at 4 °C. Coronal sections (50 µm) were cut with a vibratome. The free-floating sections were incubated in antigen retrieval solution (2.94% Tri-sodium citrate in distilled H_2_0, pH 8.5) for 20 min at 83 °C before staining. After cooling to room temperature, the sections were subsequently incubated in 50 mM glycine in PBS followed by 0.2% triton in PBS (PBST) for ten minutes each, then washed twice with 10% normal horse serum (NHS) in PBS for 30 min. The sections were incubated overnight at 4 °C with rabbit anti-Fos (Ab190289, 1:1000 in 10% NHS in PBST, Abcam, United Kingdom). The next day, after washing twice with 10% NHS, the sections were incubated with donkey anti-rabbit Alexa 488 (Ab21206, dilution 1:700 in 10% NHS in PBS, Thermo Fischer Scientific, USA) for 1 h at room temperature, then washed again and incubated in Hoechst (#H3670, 1:10 000 in PBS, Thermo Fischer Scientific, United Kingdom) for 10 min. The sections were washed in PBS and incubated for a minimum of 10 min in 10 mM TRIS–HCl before mounting with Mowiol. Specificity of the primary antibody was tested by omitting exposure of sections to the rabbit anti-Fos.

#### Fos quantification

Immunofluorescence was visualized with a laser-scanning confocal microscope (Leica TCS SP8, Germany) using identical illumination parameters for sections prepared within each staining. Z-stack images (2 µm planes) were obtained and stacked images were subsequently overlaid with the corresponding atlas details for evaluation of Fos positive counts in anatomically defined regions of interests. For quantification, an identical contrast threshold range was applied to sections from control and treated animals, using the background fluorescence intensity obtained from negative control sections (identical staining and imaging procedures but with the primary Fos antibody omitted) as the minimum intensity threshold. Positive-labelled counts (cells displaying fluorescence intensities above the minimum threshold) within the regions of interest were stereologically counted using the ImageJ software (version 1.50a, National Institutes of Health, USA) and Leica Application Suite X (Leica, Germany).

### Statistical analysis

Data are presented as mean ± standard error of the mean (S.E.M) and analyzed using GraphPad Prism (version 7.05). Data from behavioral experiments were examined either using the one-way ANOVA repeated measures with Dunnett’s, one-way ANOVA with Dunn’s or two-way ANOVA repeated measures with Tukey’s multiple comparison tests as indicated in legend text. Histological data was compared using the unpaired Student’s t-test and the Fos counts were compared using one-way ANOVA with Dunn’s multiple comparison test. A *p*-value of < 0.05 was considered significant in all tests.

## Results

### Establishing visceral hypersensitivity in DSS-induced colitis mice

**Figure 1:**
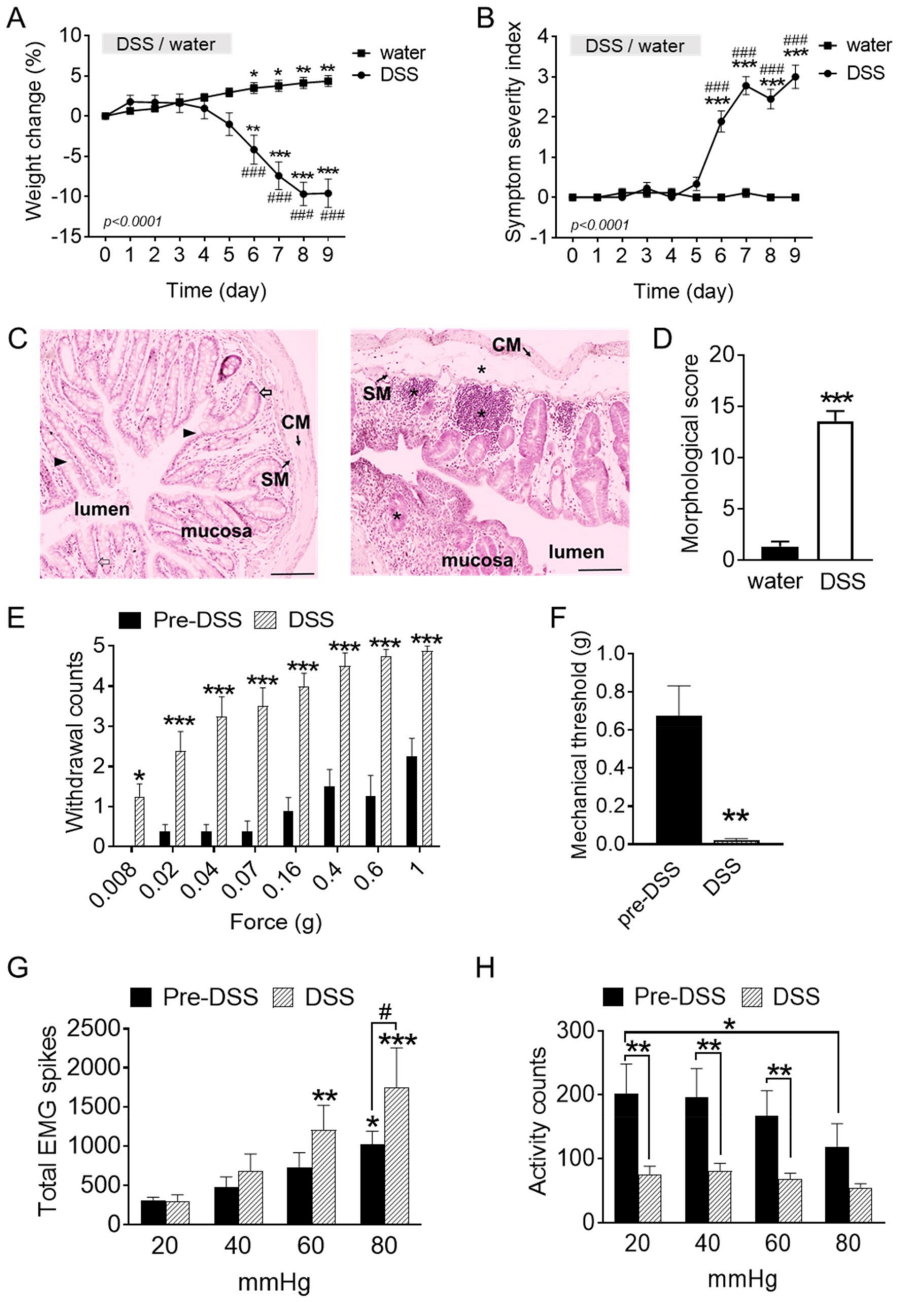
Visceral hypersensitivity in the DSS-induced colitis mice. (**A**) Gradual loss of weight and (**B**) display of disease symptoms in control (given water) and colitis (given DSS) mice (n = 8/group). DSS was given over 5 days (indicated by gray bars). (**C**) Morphological features of healthy (left) versus DSS-inflamed colon tissue (right). Extensive edema, infiltrations (indicated by *), loss of goblet cells (triangles) and crypt (open arrow) malformation are observed in the DSS animals but not in healthy animals. Scalebar, 100 µm (**D**) Quantification of morphological damage in the distal colon post-DSS compared to pre-DSS (n = 5–8/group). (**E**,**F**) Abdominal withdrawal to mechanical stimulation (0.008–1 g) is significantly increased and 40% mechanical thresholds significantly decreased in DSS-exposed mice (n = 8/group). (**G**) Total number of visceromotor responses to colorectal distension increases after DSS exposure and (**H**) activity bouts decreases after DSS exposure (n = 8/group). (**A**,**B**) *p < 0.05, **p < 0.01, ***p < 0.001 compared to respective Day 0 value and ^###^*p* < 0.001 compared between groups; (**E**) *p < 0.05, ***p < 0.001 compared to pre-DSS value; (**G**, **H**) *p < 0.05, **p < 0.01, ***p < 0.001 compared to respective 20 mmHg value and ^#^*p* < 0.05 compared between groups (indicated by corresponding line in graph), all with two-way ANOVA repeated measures with Bonferroni’s multiple comparison; in panels (**D**,**F**), **p < 0.01, ***p < 0.001, unpaired Student’s *t*-test. *p* values in panels (**A**,**B**) indicate significance between entire curves. *CM* circular muscle, *SM* submucosa.

To investigate the role of cortical activity in visceral hypersensitivity, we first established an acute DSS-induced colitis model. Here, experimental animals were treated with DSS (1.5–2%) via drinking water over 5 days. We observed that DSS-treated mice exhibited significant weight loss after DSS was withdrawn, compared to control animals that were only given normal drinking water (Day 9 DSS vs water: − 9.61 ± 1.77% vs 4.35 ± 0.69% of weight change, *p* < 0.0001; Fig. 1A). This was also accompanied by a significant increase in the symptom severity scores of these treated animals compared to their respective control group (Day 9—DSS vs water: 3 ± 0.29 vs 0 ± 0, *p* < 0.0001; Fig. 1B). Post-hoc macroscopic analysis of the colon indicated significant damage to the tissue and crypt architecture, with visible signs of edema and infiltrates (examples in Fig. 1C), which was reflected by a significantly greater morphological damage score after DSS exposure compared to non-inflamed colon sections (pre: 1.29 ± 0.29 vs post: 12.94 ± 0.94, *p* < 0.0001; Fig. 1D). These results support that our DSS treatment successfully induced pathological features commonly described in colitis^22^.

In order to examine visceral hypersensitivity, we applied von Frey filaments of increasing forces to the lower abdominal region pre-DSS (Day 0) and post-DSS (Day 6) in the same animals. We measured the filament withdrawal frequency (e.g. abdominal contraction, writhing or licking of the abdominal region), that were signs typically indicative of visceral pain. We observed that withdrawal frequencies to all mechanical stimulations applied (0.008–1 g) were significantly increased post- compared to pre-DSS (Fig. 1E), with DSS-treated animals displaying more than a twofold increase in withdrawal to 1 g stimulation (pre: 2 ± 0.5 *vs* post: 5 ± 0.1; Fig. 1E). This was also reflected in a significant drop in the 40% mechanical threshold of these animals post- compared to pre-DSS (pre: 0.71 ± 0.15 *vs* post: 0.02 ± 0.01 g, *p* = 0.004; Fig. 1F), indicating that DSS treatment indeed induced visceral hypersensitivity.

Such behavioral observations were also accompanied by a significant increase in abdominal contractions to CRDs applied in vivo. Using wireless telemetric sensors, we examined the visceromotor responses (VMRs) by assessing the total EMG spikes recorded from the lower abdominal regions of the freely-moving mice in response to graded CRDs (20, 40, 60, 80 mmHg). Under normal non-inflamed conditions, increases in VMRs were observed upon applications of increasing CRD pressures, with significantly higher VMRs recorded during noxious compared to non-noxious CRD intensities (20 mmHg: 308 ± 41 vs 80 mmHg: 1017 ± 168, *p* = 0.046; Fig. 1G).

After DSS treatment, in the same mice, we observed a similar trend with greater VMRs recorded, particularly at higher intensities of CRDs (60 and 80 mmHg), compared to 20 mmHg. Additionally, noxious 80 mmHg CRD induced a significantly greater abdominal contractile activity post-DSS treatment (pre: 1017 ± 168 *vs* post: 1743 ± 511, *p* = 0.026; Fig. 1G). In freely-moving animals, abdominal contractions perceived as painful are typically also reflected by inactivity or immobility. Here, telemetric recordings of the total activity bouts during the in vivo CRDs were observed to be significantly decreased in non-inflamed animals only during noxious CRD applications (20 mmHg: 202 ± 47 vs 80 mmHg: 119 ± 36, *p* = 0.022; Fig. 1H). This loss of activity was significantly more profound after DSS treatment in response to all CRD pressures applied (Fig. 1H).

### Cortical activation patterns induced by noxious and non-noxious mechanical CRD stimulation

**Figure 2:**
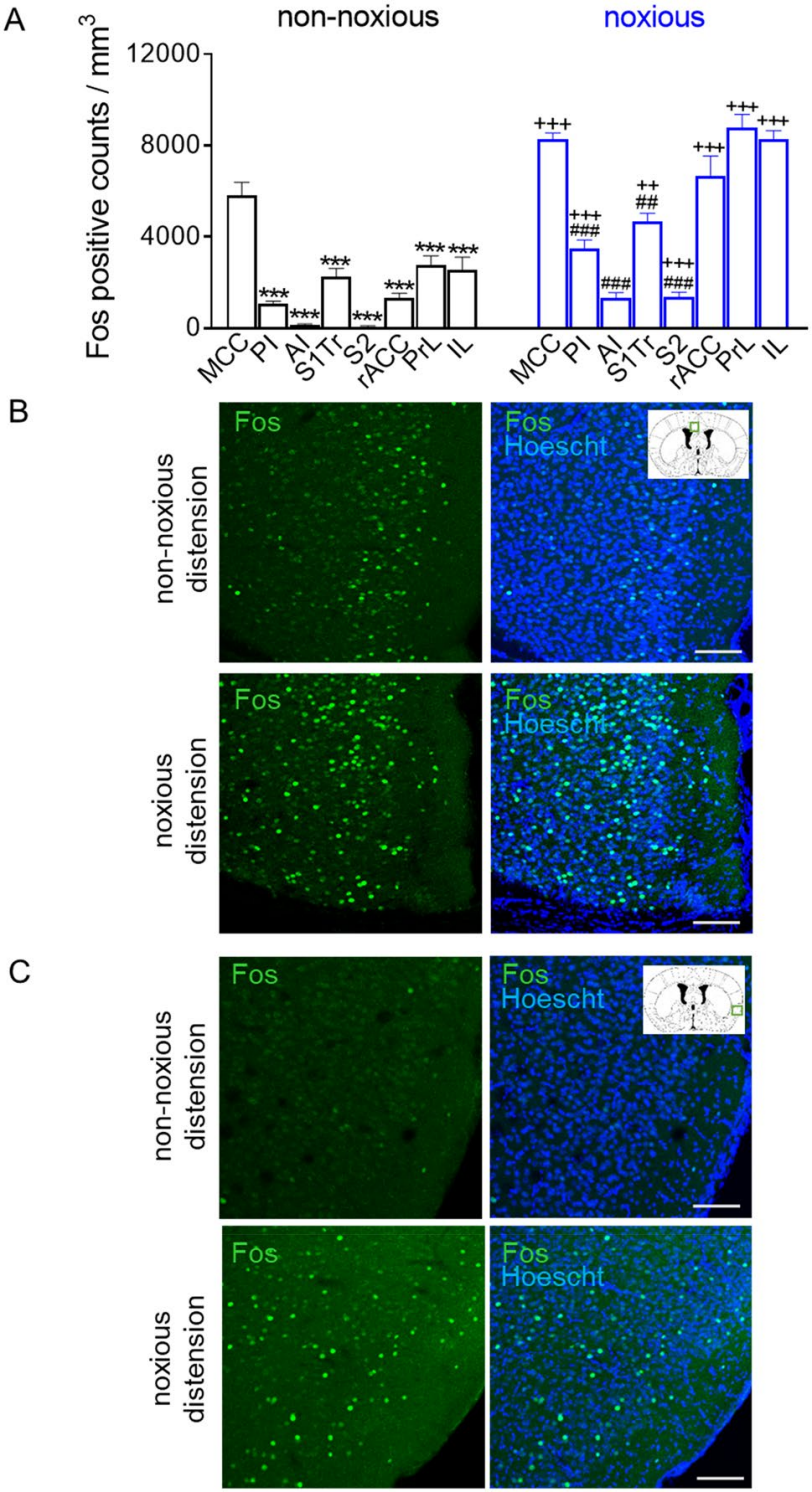
Cortical activation patterns to non-noxious and noxious colorectal distension applied in vivo. (**A**) Quantification of the Fos positive counts in several cortical regions after non-noxious colorectal distension (black) and after noxious colorectal distension (blue). Fos expression was also upregulated after noxious colorectal distension within these regions (**B**,**C**) Example images of Fos (green) and Hoescht (blue) expression in the (**B**) MCC and (**C**) PI. Scale bar, 100 µm. ***p < 0.001 compared to MCC (non-noxious); ^##^*p* < 0.01, ^###^*p* < 0.001 compared to MCC (noxious); ^++^*p* < 0.01, ^+++^*p* < 0.001 compared with corresponding non-noxious group, all with one-way ANOVA with Dunn ‘s multiple comparison; *MCC* midcingulate cortex, *PI* posterior insula, *AI* anterior insula, *S1Tr* primary somatosensory trunk region, *S2* secondary somatosensory, *rACC* rostral anterior cingulate cortex, *PrL* prelimbic cortex, *IL* infralimbic cortex.

In order to elucidate the cortical regions that are involved in the processing of visceral nociception during acute colitis, we first compared the Fos expression levels (marker for neuronal activation) in several sensory and associative cortical areas in non-inflamed mice that were given either a noxious (80 mmHg) or non-noxious (20 mm Hg) CRD mechanical stimulation. Here, we focused on the mid-cingulate cortex (MCC), anterior (AI) and posterior insula (PI), somatosensory cortex (trunk region; S1Tr), secondary somatosensory cortex (S2) as well as prefrontal prelimbic (PrL), infralimbic (IL) and the rostral anterior cingulate cortices (rACC). We observed a significant Fos upregulation in the MCC in response to noxious stimuli compared to non-noxious stimuli applications (noxious: 8276 ± 269 vs non-noxious: 5797 ± 591 counts/mm^3^, *p* = 0.0009; Fig. 2A, examples in Fig. 2B). Similarly, significantly elevated Fos levels were also detected in the PI (noxious: 3471 ± 391 vs non-noxious: 1070 ± 109 counts/mm^3^, *p* < 0.0001; Fig. 2A, examples in Fig. 2C).

A comparison of the upregulated Fos expression levels indicated significant increases in neuronal activity in numerous cortical regions examined after noxious CRD compared to non-noxious CRD (Fig. 2A); in the S1Tr (noxious: 4661 ± 364 *vs* non-noxious: 2258 ± 357 counts/mm^3^, *p* = 0.003), AI (noxious: 1324 ± 227 vs non-noxious: 156 ± 28 counts/mm^3^, *p* < 0.0001), in the S2 (noxious: 1379 ± 198 *vs* non-noxious: 69 ± 21 counts/mm^3^, *p* < 0.0001) as well as in the PrL (noxious: 8775 ± 579 vs non-noxious: 2777 ± 384 counts/mm^3^, *p* < 0.0001), IL (noxious: 8281 ± 372 vs non-noxious: 2549 ± 560 counts/mm^3^, *p* < 0.0001) and rACC (noxious: 6661 ± 870 vs non-noxious: 1379 ± 198 counts/mm^3^, *p* < 0.0001). Interestingly, amongst these regions examined, we consistently observed a significantly greater activation in the MCC areas compared to all other examined cortical regions, in response to both non-noxious and noxious CRDs applied in vivo (Fig. 2A). These results suggest that firstly, there is a widespread activation of cortical networks that become increasingly recruited during the processing of noxious visceral stimuli and likely contribute to visceral nociception. Secondly, our data show that whilst the S1Tr, MCC, PI and prefrontal cortices are important for the induction of visceral behavioral hypersensitivity from intense nociceptive stimuli, ongoing neuronal activity in the MCC, in particular, appears to majorly participate in the modulation of both innocuous and noxious visceral sensory inputs.

### Suppression of MCC activity alleviates colitis-induced mechanical hypersensitivity and anxiety via specific cortico-cortical modulation

Based on the observations above, we further investigated if induction of behavioral plasticity during acute colitis is modulated by ongoing activity in the MCC. To test this, we used a chemogenetic approach using rAAV delivery of an inhibitory DREADD, namely the human M4 muscarinic receptor (hM4Di), that can be activated by the inert clozapine metabolite clozapine-N-oxide (CNO)^26^. Activation of the hM4Di in neurons engages the Gi signaling pathway in neurons to subsequently decrease neuronal firing rates.

**Figure 3:**
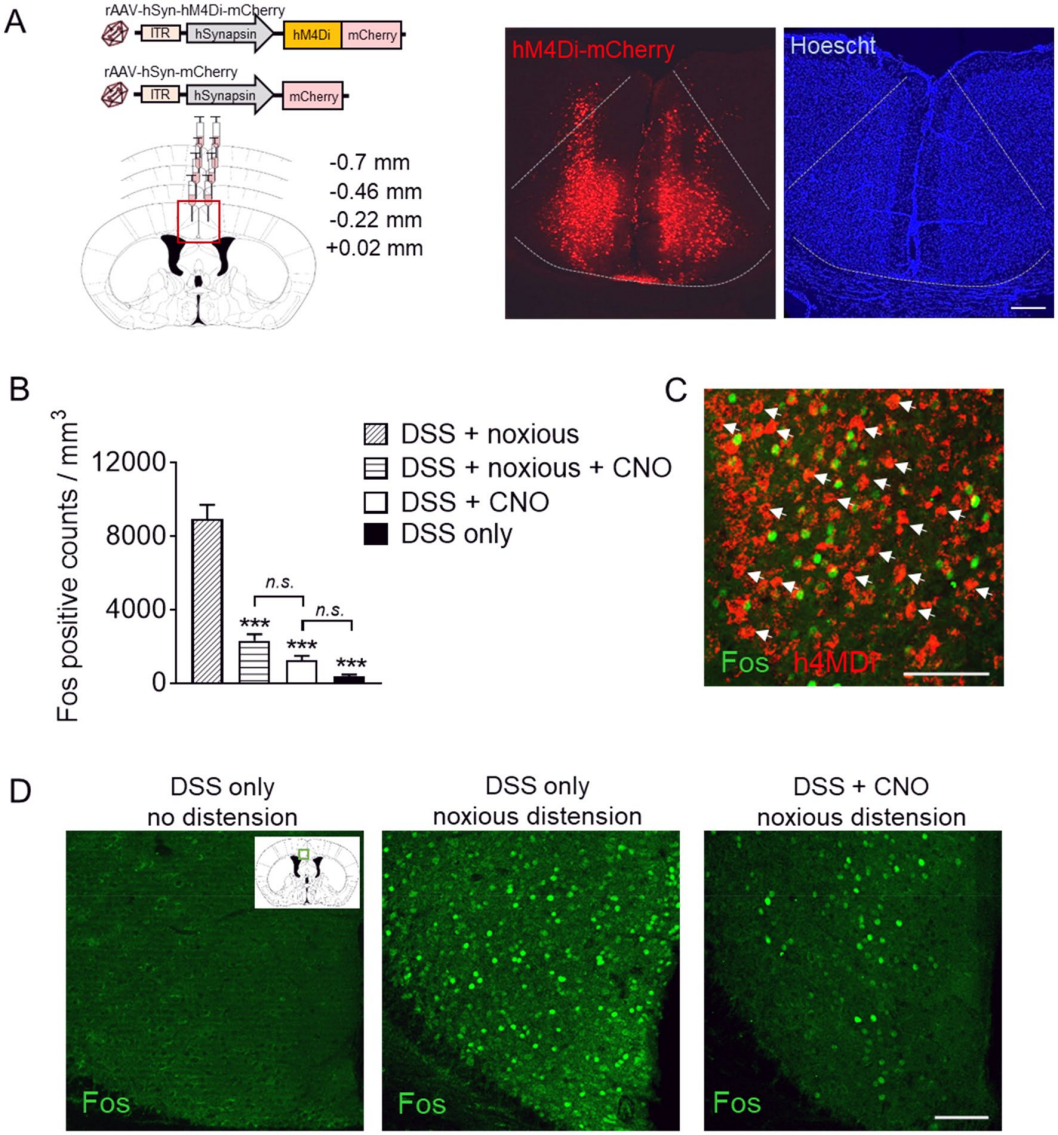
Chemogenetic targeting of MCC activity during experimental colitis. (**A**) Experimental scheme (left) and representative images (right) of DREADD targeting in the bilateral MCC. (**B**) Quantification of Fos positive counts in the MCC in response to noxious CRD (80 mmHg) in the absence or presence of DSS and CNO (*n* = 20–28 sections/group from 3 mice). (**C**) Representative example of non-overlapping hM4Di-expressing cells (red) and Fos expression (green, indicated by arrows). (**D**) Examples of Fos expression (green) in hM4Di-expressing animals under different DSS conditions. Scale bars, 200 µm (**A**), 100 µm (**C**,**D**). ***p < 0.001 compared to DSS + noxious, one-way ANOVA with Dunn’s multiple comparison test; *n.s.* not significant.

In order to achieve infection of the entire MCC region, we carried out multiple stereotactic injections of rAAV particles to induce expression of either mCherry only or mCherry-tagged hM4Di in the MCC in a highly efficient manner using a synapsin promoter (scheme and example in Fig. 3A). To validate the DREADD in vivo, we analyzed c-Fos expression levels in the MCC in response to noxious CRD in the absence and presence of CNO. Here, analysis of c-Fos expression indicated a significant reduction in the overall MCC activation levels in hM4Di-expressing DSS-treated mice that were injected with CNO followed by noxious CRDs, compared to animals that only received noxious CRDs, demonstrating that chemogenetic suppression could effectively reduce MCC neuronal activation induced by noxious visceral mechanical stimulation in the colitis animals (DSS + noxious CRDs: 8933 ± 785 vs DSS + noxious CRDs + CNO: 2326 ± 342 counts/mm^3^, *p* = 0.0009; Fig. 3B, examples in Fig. 3D). Furthermore, no differences were observed between DSS-treated animals that were given only CNO or given CNO followed by noxious CRDs (DSS + noxious CRD + CNO: 2326 ± 342 vs DSS + CNO: 1243 ± 249 counts/mm^3^, *p* > 0.999). Although DSS animals given only CNO showed a trend for upregulated MCC activity compared to home cage DSS animals, this did not reach significance (DSS + CNO: 1243 ± 249 vs DSS only: 374 ± 101 counts/mm^3^, *p* = 0.49; Fig. 3B, examples in Fig. 3D). In DSS animals that received CNO, we routinely observed that the majority of the hM4Di-mCherry labelled cells did not co-express with Fos (Fig. 3C).

**Figure 4:**
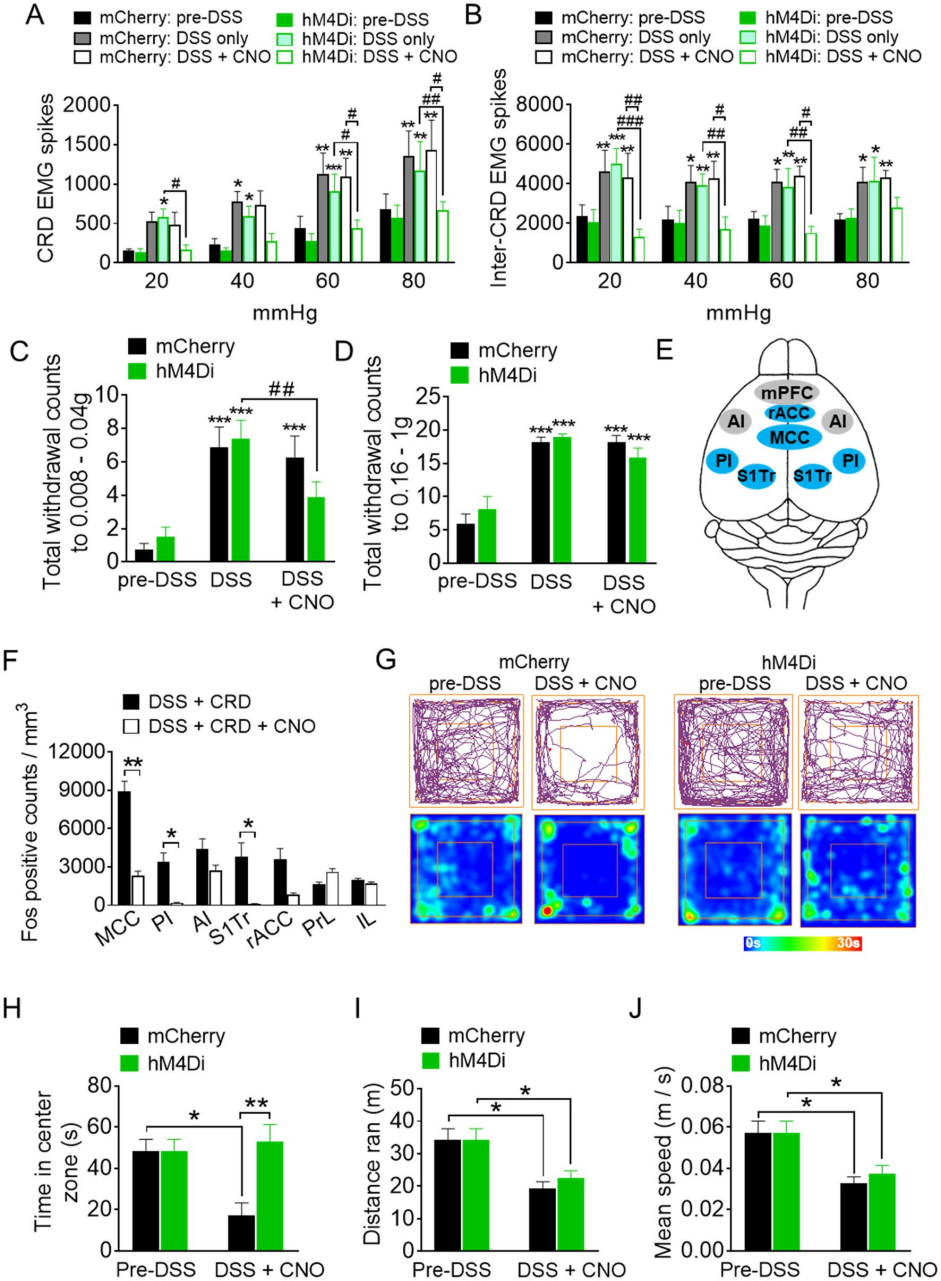
Behavioral and visceromotor responses to functional suppression of MCC activity. (**A**) Total number of visceromotor responses during colorectal distension (CRD) is attenuated during MCC suppression in hM4Di-expressing DSS animals and (**B**) total number of visceromotor responses during inter-CRD periods is attenuated during MCC suppression in hM4Di-expressing DSS animals (same n = 5–6/group in (**A**,**B**)). (**C**,**D**) Withdrawal counts to low intensity (in **C**) and high intensity (in **D**) mechanical forces applied to the abdominal skin (same n = 8/group in **C,D**). (**E**) Scheme depicting changes in cortical excitability from MCC activity suppression. Blue and gray regions indicate significantly reduced or unaffected activity, respectively. (**F**) Quantification of Fos levels (from panel **E**) in hM4Di-expressing DSS mice after noxious CRD applications (with and without CNO; *n* = 15–25 sections/group from 3 mice). (**G**) Tracking plot (upper row) and time map (lower row) examples (obtained from ANY-maze software version 6.1, http://www.anymaze.co.uk) of mCherry- and hM4Di-expressing animals performing the open field exploratory task before and after DSS treatment (post-CNO). Quantification of the time spent in the center open area (**H**), total distance ran (**I**) and mean speed (**J**) of mCherry and hM4Di groups before and after DSS treatment (post-CNO) (n = 8/group in **H**–**J**). In panels (**A**–**C**), *p < 0.05, **p < 0.01, ***p < 0.001, two-way repeated ANOVA with Tukey’s compared to respective pre-DSS values and ^*#*^*p* < 0.05, ^*##*^*p* < 0.01, two-way repeated ANOVA with Tukey’s compared between groups (indicated by corresponding line in graph); in panels (**F**,**H–J**), *p < 0.05, **p < 0.01, one-way ANOVA with Dunn’s multiple comparison test. *MCC* midcingulate cortex, *PI* posterior insula, *AI* anterior insula, *S1Tr* primary somatosensory (trunk) cortex, *rACC* rostral anterior cingulate cortex, *PrL* prelimbic cortex, *IL* infralimbic cortex, mPFC medial prefrontal cortex (PrL and IL).

Assessment of the VMRs demonstrated that whilst inflamed animals showed significant enhanced VMRs to CRDs applied in vivo, after CNO injections, only the hM4Di-expressing mice showed significant attenuation in VMRs during 20, 60 and 80 mmHg CRD applications (light green and open green bars in Fig. 4A). Moreover, the number of recorded VMRs in these animals were also significantly lower compared to the mCherry animals, particularly during high intensity CRD applications (open black and open green bars in Fig. 4A). The VMR levels observed between the CRD applications (inter-CRD stimulus) were also significantly reduced in the hM4Di-expressing animals after receiving CNO, suggesting that spontaneous abdominal contractility was additionally attenuated upon inhibition of MCC activity (Fig. 4B).

When we examined the mechanical sensitivity of the abdominal region of these animals, we observed that both the mCherry- and hM4Di-expressing groups of animals exhibited a significant increase by almost fourfold in the total number of withdrawals to a range of low and high mechanical forces applied to the visceral skin after DSS treatment (Fig. 4C,D), indicating visceral allodynia and hyperalgesia were indeed induced by colon inflammation. When we assessed the mechanical sensitivity 1 h after CNO was injected, the hM4Di-expressing DSS mice showed a significant attenuation in abdominal withdrawal to low intensities of mechanical forces after CNO was injected (DSS pre-CNO: 7.0 ± 1 vs DSS post-CNO: 4 ± 1 withdrawal counts, *p* = 0.004; Fig. 4C), which was absent in the mCherry control group (DSS pre-CNO: 7 ± 1 vs DSS post-CNO: 6 ± 1, *p* = 0.8; Fig. 4C). Visceral mechanical hyperalgesia to high mechanical forces was unaffected by suppression of MCC activity in both the mCherry or hM4Di-expressing DSS animals (Fig. 4D).

In order to examine the nature of MCC suppression on cortical network changes that alleviated visceral mechanical hypersensitivity, we analyzed and compared the c-Fos expression patterns in the cortex of DSS-treated animals that received noxious CRDs without and with MCC suppression. Here, we observed that MCC silencing led to decreased excitability in the PI, S1Tr and rACC (highlighted in blue, Fig. 4E, quantification in Fig. 4F), but had no effects on the prefrontal regions such as the PrL, IL and AI excitability (highlighted in gray, Fig. 4E, quantification in Fig. 4F).

To assess effects of MCC suppression on colitis-induced anxiety-related behavior, we carried out the open field novel exploratory test, which is one of the most commonly used behavioral paradigm to evaluate an animal’s emotional state. In this assay, the tendency to avoid exploring or running in the open center space (i.e. potentially dangerous) has been validated as a measure of anxiogenic behavior in mice^27^. We observed that after administering CNO, DSS-treated hM4Di-expressing mice spent a significantly greater amount of time exploring the open unprotected center zone compared to DSS-treated mCherry controls (examples in Fig. 4G; mCherry: 17.0 ± 6.2 vs hM4Di: 53.0 ± 8.1 s, *p* = 0.006; Fig. 4H). Furthermore, this exploration time was similar to levels observed in normal non-inflamed hM4D-expressing mice (pre-DSS: 48.3 ± 5.7 s, *p* = 0.95; Fig. 4H), suggesting that inhibition of MCC activity attenuated anxiety-like behavior induced by colitis. The anxiolytic effect brought about by MCC suppression in the DSS-treated animals was not likely due to disturbances in motor function and locomotion as no differences in distance and speed parameters were found between mCherry and hM4Di animals (Fig. 4I,J).

## Discussion and conclusions

Our present study is the first to report a causal link between a specific cingulate domain, namely the MCC and visceral hypersensitivity generated by intestinal inflammation. Clinical studies employing neuroimaging techniques frequently describe abnormalities in evoked responses, altered resting-state activity and connectivity as well as structural remodeling of the cingulate cortices in IBD and irritable bowel syndrome patients^16,28–31^, highlighting that significant plasticity in these cingulate regions, which are key components of pain, emotion and homeostatic networks, are likely a contributing factor to the commonly reported higher levels of pain and mood disorders in these patients. However, the inability to modulate the activity of defined neuronal populations underlying pain processing remains a major technical limitation of clinical studies. Surprisingly, despite the amount of available clinical evidence, targeted preclinical investigations of the contributions of cingulate subdomains in visceral pain have not been previously carried out. This validation in animal models is important in order to provide mechanistic information that can aid new cingulate-mediated therapies^32^, an emerging treatment strategy for pain and psychiatric disorders arising from gastrointestinal diseases which currently remain undertreated and undermanaged.

Previous work has been undertaken to examine brain areas involved in processing physiological non-noxious and noxious mechanical distension of the colon, although these studies have either focused on activation patterns within subcortical and brain stem regions or examined the cortical areas only in response to noxious visceral inputs^33–35^. In our present study, detailed molecular analysis of c-Fos expression levels showed that within the rodent cortex, the MCC in particular, was consistently highly activated in response to both non-noxious and noxious visceral stimuli under non-inflamed gut conditions, in comparison to the somatosensory, insula, rACC or prefrontal cortices. These initial findings supported our further interrogative analyses using a chemogenetic approach to functionally manipulate the ongoing output activity of the entire bilateral MCC, in order to address its role in behavioral changes induced by acute experimental colitis. Our results show that the MCC, is indeed important for induction of visceral hypersensitivity as well as anxiety-like phenotype associated with gut inflammation.

A recent functional imaging study in another animal model of colitis has also observed a strong activation of the MCC in response to noxious CRD^36^, albeit activity in the MCC became markedly enhanced in response to noxious CRD only after the induction of gut inflammation. This was not apparent in our current c-Fos expression analyses where our results indicate that nociceptive visceral input strongly activates the MCC in both the absence and presence of gut inflammation. The lower cellular resolution of imaging tools in addition to the state of the animals during CRD (sedated versus conscious freely-moving in our study) likely account for the differences reported. In our previous study, we reported that in vivo silencing of MCC activity using optogenetic tools led to an attenuation of peripheral inflammation-induced mechanical hypersensitivity of the injected hind paw (with Complete’s Freund adjuvant)^19^. More recently, Hu and colleagues discovered that projections arising from subregions of the MCC could selectively modulate neuropathic pain^18^, further supporting our current findings that the MCC could indeed be an important cortical hub that is widely recruited during pathological pain arising from peripheral inflammation and injuries.

To our knowledge, this study is the first to use chemogenetic approaches to investigate cortical plasticity underlying visceral pain during gut inflammation. This technology represents a useful tool for reversible, non-invasive short- or long-term manipulation of activity within targeted brain regions that can aid our understanding of visceral pain syndromes. Our results reveal that blocking activity in the MCC during colitis prevented the development of visceral mechanical hypersensitivity. Further c-Fos-based activity mapping of cortical excitability patterns indicated that the alleviation of visceral hypersensitivity by MCC inhibition arose from a downstream suppression of sensitization amongst brain regions including the posterior insula and somatosensory cortex (trunk region), areas that receive incoming nociceptive inputs from the gut via thalamic projections and are associated with the sensory-discriminative cortical network. This finding additionally supports our previous report of a direct MCC to posterior insula afferent pathway that functionally induces and maintains peripheral nociceptive hypersensitivity in the hind paws^19^. Notably, we found a functional association between the MCC and the rostral anterior cingulate cortex (rACC), which has not been previously identified. Synaptic plasticity in the ACC, has been causally linked to pain and pain-associated emotional dysfunction such as anxiety, depression and aversion^37–40^. In particular, it was recently reported that the co-existence of anxiety in IBD patients, was a predictive factor for a higher risk of a poorer disease outcome^41^. As we observed that inhibition of activity in the MCC led to a reduced rACC excitability, we undertook the open field exploratory test to investigate if functional suppression of the MCC could additionally lead to an anxiolytic effect in our colitis mice. Indeed, our results indicated that anxiety-like behavior during experimental colitis could be alleviated by reducing MCC activity, possibly via a downstream disruption of rACC excitability. As we did not observe changes in c-Fos levels in the prefrontal regions after MCC activity suppression, it is unlikely that the anxiolytic effect we observed was due to modulation by the prelimbic or infralimbic cortices that have been previously described to modulate anxious states in other pain models^42–44^. Furthermore, it is also well-known that stress is a trigger factor that is associated with the onset and exacerbation of symptoms of bowel diseases^45,46^ through dysregulation of emotional and arousal central circuits that activate the hypothalamic–pituitary–adrenal axis (HPA) and descending aminergic pathways. It is possible that the impaired top-down input from the MCC to these systems via the rACC may have also contributed to the dampened colitis-induced hypersensitivity and anxiety observed in our study.

Since both stress and anesthesia majorly influence peripheral inflammation and visceral nociceptive responses, in our present study, we adapted a more physiologically-relevant, unrestrained conscious recording environment by using subcutaneous telemetry sensors that are chronically implanted in our animals. To date, most conventional studies assessing VMR to visceral distension in animal models have mostly relied on sutured abdominal electrode wires that protrude from the neck and are attached to external computer cables during the experiment, or are carried out in restraint, wrapped or lightly anaesthetized animals. Our telemetry system allows continuous monitoring of EMG responses in our mice in a freely-moving, conscious and restraint-free state in their home cages. Previous groups have also used similar telemetry systems and found them to be an adequate tool to assessing muscle EMGs in visceral models^47–49^.

We acknowledge several limitations in our present study. Firstly, given that the goal of this study was to investigate the effects of short-term acute colitis on brain activity and behaviors, only male mice were used as they have been previously reported to develop quicker and more aggressive colitis than females^5,22^. Future studies, especially chronic longitudinal studies, would need to additionally employ female animals to compare for mechanistic differences between the development and maintenance of visceral hypersensitivity and comorbid behaviors. Secondly, as we were interested in achieving a functional suppression of the overall MCC activity output, we had infected all neuronal populations within the MCC with our chemogenetic approach. Post-hoc analyses with Fos as our molecular marker for neuronal activation indicated that we could successfully reduce the entire MCC excitability level after activation of the inhibitory DREADD in vivo. Future investigations of a detailed dissection of the contributions of inhibitory and excitatory populations as well as cortical layer contribution and their consequent targets would be needed to narrow down additional key contributors of the phenomena we observed in this study. Such work would shed further light on the causal underpinnings of visceral pain reported in remissive IBD patients (in the absence of gut inflammation or obvious pathology), which is the major debilitating factor for these sufferers.

In conclusion, acute intestinal inflammation provokes a state of visceral hypersensitivity that is markedly attenuated by functionally disrupting activity in the MCC. Targeting MCC plasticity in early stages of colitis may prevent the onset and/or exacerbation of behavioral changes that frequently occur in IBDs. These results provide a mechanistic insight to the communication within the gut-brain axis, which will be important for therapeutic considerations for the treatment of pain and psychiatric comorbidities in IBD.

## References

[1] Burisch J, Jess T, Martinato M, Lakatos PL (2013). The burden of inflammatory bowel disease in Europe. J. Crohns Colitis.

[2] Bonaz BL, Bernstein CN (2013). Brain-gut interactions in inflammatory bowel disease. Gastroenterology.

[3] Neuendorf R, Harding A, Stello N, Hanes D, Wahbeh H (2016). Depression and anxiety in patients with Inflammatory Bowel Disease: a systematic review. J. Psychosom Res..

[4] Bernstein CN (2019). Increased burden of psychiatric disorders in inflammatory bowel disease. Inflamm. Bowel Dis..

[5] Nyuyki KD, Cluny NL, Swain MG, Sharkey KA, Pittman QJ (2018). Altered brain excitability and increased anxiety in mice with experimental colitis: consideration of hyperalgesia and sex differences. Front. Behav. Neurosci..

[6] Jain P (2015). Behavioral and molecular processing of visceral pain in the brain of mice: impact of colitis and psychological stress. Front. Behav. Neurosci..

[7] Russo R (2018). Gut-brain axis: role of lipids in the regulation of inflammation, pain and CNS diseases. Curr. Med. Chem..

[8] Sochocka M (2019). The gut microbiome alterations and inflammation-driven pathogenesis of Alzheimer's disease—a critical review. Mol. Neurobiol..

[9] Sampson TR (2016). Gut microbiota regulate motor deficits and neuroinflammation in a model of Parkinson's disease. Cell.

[10] Zonis S (2015). Chronic intestinal inflammation alters hippocampal neurogenesis. J. Neuroinflamm..

[11] Do J, Woo J (2018). From gut to brain: alteration in inflammation markers in the brain of dextran sodium sulfate-induced colitis model mice. Clin. Psychopharmacol. Neurosci..

[12] Han Y (2018). Cortical inflammation is increased in a DSS-induced colitis mouse model. Neurosci. Bull..

[13] Sroor HM (2019). Experimental colitis reduces microglial cell activation in the mouse brain without affecting microglial cell numbers. Sci. Rep..

[14] Straub RH, Herfarth H, Falk W, Andus T, Schölmerich J (2002). Uncoupling of the sympathetic nervous system and the hypothalamic–pituitary–adrenal axis in inflammatory bowel disease?. J. Neuroimmunol..

[15] Mearin F (2016). Bowel disorders. Gastroenterology.

[16] Mayer EA, Gupta A, Kilpatrick LA, Hong JY (2015). Imaging brain mechanisms in chronic visceral pain. Pain.

[17] Bao C (2017). Differences in brain gray matter volume in patients with Crohn's disease with and without abdominal pain. Oncotarget.

[18] Hu TT (2019). Activation of the intrinsic pain inhibitory circuit from the midcingulate Cg2 to zona incerta alleviates neuropathic pain. J. Neurosci..

[19] Tan LL (2017). A pathway from midcingulate cortex to posterior insula gates nociceptive hypersensitivity. Nat. Neurosci..

[20] Kragel PA (2018). Generalizable representations of pain, cognitive control, and negative emotion in medial frontal cortex. Nat. Neurosci..

[21] Vogt BA (2016). Midcingulate cortex: structure, connections, homologies, functions and diseases. J. Chem. Neuroanat..

[22] Chassaing B, Aitken JD, Malleshappa M, Vijay-Kumar M (2014). Dextran sulfate sodium (DSS)-induced colitis in mice. Curr. Protoc. Immunol..

[23] Eichele DD, Kharbanda KK (2017). Dextran sodium sulfate colitis murine model: an indispensable tool for advancing our understanding of inflammatory bowel diseases pathogenesis. World J. Gastroenterol..

[24] Paxinos G, Franklin KBJ (2001). The Mouse Brain in Stereotaxic Coordinates.

[25] Kim JJ, Shajib MS, Manocha MM, Khan WI (2012). Investigating intestinal inflammation in DSS-induced model of IBD. J. Vis. Exp..

[26] Roth BL (2016). DREADDs for neuroscientists. Neuron.

[27] La-Vu M, Tobias BC, Schuette PJ, Adhikari A (2020). To approach or avoid: an introductory overview of the study of anxiety using rodent assays. Front. Behav. Neurosci..

[28] Agostini A (2017). Stress and brain functional changes in patients with Crohn's disease: a functional magnetic resonance imaging study. Neurogastroenterol. Motil..

[29] Naliboff BD (2001). Cerebral activation in patients with irritable bowel syndrome and control subjects during rectosigmoid stimulation. Psychosom Med..

[30] Agostini A (2013). New insights into the brain involvement in patients with Crohn's disease: a voxel-based morphometry study. Neurogastroenterol. Motil..

[31] Bao CH (2015). Alterations in brain grey matter structures in patients with Crohn's disease and their correlation with psychological distress. J. Crohns Colitis.

[32] Vogt BA (2013). Inflammatory bowel disease: perspectives from cingulate cortex in the first brain. Neurogastroenterol. Motil..

[33] Mönnikes H (2003). Differential induction of c-fos expression in brain nuclei by noxious and non-noxious colonic distension: role of afferent C-fibers and 5-HT3 receptors. Brain Res..

[34] Traub RJ, Silva E, Gebhart GF, Solodkin A (1996). Noxious colorectal distention induced-c-Fos protein in limbic brain structures in the rat. Neurosci. Lett..

[35] Wang L, Martínez V, Larauche M, Taché Y (2009). Proximal colon distension induces Fos expression in oxytocin-, vasopressin-, CRF- and catecholamines-containing neurons in rat brain. Brain Res..

[36] Huang T (2019). Pain matrix shift in the rat brain following persistent colonic inflammation revealed by voxel-based statistical analysis. Mol. Pain.

[37] Meda KS (2019). Microcircuit mechanisms through which mediodorsal thalamic input to anterior cingulate cortex exacerbates pain-related aversion. Neuron.

[38] Zhuo M (2016). Neural mechanisms underlying anxiety-chronic pain interactions. Trends Neurosci..

[39] Johansen JP, Fields HL (2004). Glutamatergic activation of anterior cingulate cortex produces an aversive teaching signal. Nat. Neurosci..

[40] Barthas F (2015). The anterior cingulate cortex is a critical hub for pain-induced depression. Biol. Psychiatry.

[41] Narula N (2019). Anxiety but not depression predicts poor outcomes in inflammatory bowel disease. Inflamm. Bowel Dis..

[42] Wang GQ (2015). Deactivation of excitatory neurons in the prelimbic cortex via Cdk5 promotes pain sensation and anxiety. Nat. Commun..

[43] Pati S, Sood A, Mukhopadhyay S, Vaidya VA (2018). Acute pharmacogenetic activation of medial prefrontal cortex excitatory neurons regulates anxiety-like behaviour. J. Biosci..

[44] Liang HY (2020). nNOS-expressing neurons in the vmPFC transform pPVT-derived chronic pain signals into anxiety behaviors. Nat. Commun..

[45] Chang L (2011). The role of stress on physiologic responses and clinical symptoms in irritable bowel syndrome. Gastroenterology.

[46] Mayer EA (2000). The neurobiology of stress and gastrointestinal disease. Gut.

[47] Nijsen MJ, Ongenae NG, Coulie B, Meulemans AL (2003). Telemetric animal model to evaluate visceral pain in the freely moving rat. Pain.

[48] Meile T, Zittel TT (2002). Telemetric small intestinal motility recording in awake rats: a novel approach. Eur. Surg. Res..

[49] Wang Z (2008). Regional brain activation in conscious, nonrestrained rats in response to noxious visceral stimulation. Pain.

